# Copper Recovery From Ammonia Solutions Through Electro-Electrodialysis (EED)

**DOI:** 10.3389/fchem.2020.622611

**Published:** 2021-01-28

**Authors:** Belen Garrido, Gerardo Cifuentes, Pedro Fredes, Eduardo Pino, Cristian Calderón, Magdalena Cifuentes-Cabezas

**Affiliations:** ^1^ Metallurgical Engineering Department, University of Santiago of Chile (USACH), Santiago, Chile; ^2^ Department of Environmental Science, University of Santiago of Chile (USACH), Santiago, Chile; ^3^ University Research Institute for Industrial, Radiophysical and Environmental Safety (ISIRYM), Universitat Politècnica de València, Valencia, Spain

**Keywords:** electro-electro dialysis, copper electrowinning, ammoniacal complexing agent, cupric tetramine, cationic ion exchange membrane

## Abstract

Alkaline leaching with highly selective ammoniacal complexing agents is an interesting alternative for the treatment of copper concentrates. This treatment is beneficial for copper recovery because it allows the formation of soluble amines complexes, with cupric tetramine ($\text{ Cu}{\left({\text{NH}}_{3}\right)}_{4}^{2+}$) being the most stable. In order to suppress the unit operation of solvent extraction (SX) and move directly to the electrochemical process, an electro-electrodialysis (EED) process using ion exchange membranes to obtain copper is proposed. The study contemplates the operation with synthetic ammonia solutions containing copper at different concentrations and current density under standard conditions of pressure and temperature. The presented data demonstrate that the concentration of copper in the solution and the excess of ammonia are inversely related to the efficiency of the current and the voltage of the cell, whereas an increase in current density causes an increase in current efficiency, contrary to what happens in sulfuric systems.

## Introduction

On leaching process of oxide and sulfured copper ores, sulfuric acid and ferric sulfate are common reagents used as oxidizing media. Within the alternatives to the treatment of sulfured copper concentrates are basic or alkaline leaching with highly selective ammoniacal complexing agents for benefit of Copper. Thus, leaching with aqueous dissolutions containing ammonium ions being supplied to the system as ammonium hydroxide, ammonium chloride, and/or ammonium carbonate (Alguacil, 1999; Ammann and Cook, 1977; Guo and Searson, 2010; Majima, 1993; Ordoñez and Alfaro, 2011; González Mercado et al., 2015) allows the formation of soluble amine complexes, and the region where the $\text{Cu}{\left({\text{NH}}_{3}\right)}_{4}^{2+}$ complex is stable can be defined as a function of temperature, total ammonia concentration ([NH_3_]+[NH_4_^+^]), and, particularly, pH. For example, in ammonium carbonate medium, the optimum pH range for stable copper complex formation is found to be between 8.3 and 10.8 (Bingöl et al., 2005), with cupric tetramine being the most stable at pH > 9.5. From these considerations, the system cannot operate at pH values higher than 9.8, due to the precipitation of tenorite (CuO) according to electrode potential vs. pH equilibria diagrams (Pourbaix diagram).

On the other hand, iron is hydrolyzed to an insoluble hydrated iron oxide, which remains on the leaching waste as a precipitate (García and Rodríguez, 2005; Pradhan et al., 2009; Ekmekyapar et al., 2012) being then removed from the system, providing a low level of impurities on the pregnant leach solution (PLS). This solution is then taken to solvent extraction (SX) and through the addition of sulfuric acid as eluent, copper sulfate is obtained to achieve operation in the conventional electrowinning cells (EW). The problem associated with this drastic change in acidity leads to acid and ammonium entrainment in the solutions charged and discharged in SX, preventing an efficient transfer of the charged solution to the electrolytic tank house at the same time that ammonium gases start to build up. This causes that processes of leaching based on ammonia not to be used, and, therefore, the possibility of obtaining a copper-rich electrolyte is lost.

In a conventional EW process, it is not possible to use directly the PLS solution from leaching due to the high concentration of copper required (>40 g·dm-3). Studies about direct electrochemical recovery of copper from ammonia solutions have been carried out with promising results (Majima, 1993; Bingöl et al., 2005; Guo and Searson, 2010; Ordoñez and Alfaro, 2011; González Mercado et al., 2015). However, there are no studies focused on toxicity and/or corrosivity issues due to the secondary reactions and attributing to the loss of ammonia to evaporation and oxidation processes, preventing its reuse in the leaching process. Therefore, electrodialysis process in closed cells is a viable alternative to conventional EW, since concentration of ionic copper in solution is not a limiting variable due to the high velocity of the solutions inside of each anodic and cathodic compartment of the closed cell. The electro-electrodialysis (EED) is a technology that uses ion exchange membranes for ion concentration and at the same time to produce electrochemical reactions at the electrodes using a continuous electric field between the electrodes inside the cell. From this, it is possible to obtain species of commercial value in the electrolytes and at the electrodes. The EED cells are composed of two electrodes, cathode and anode, separated by an ionic membrane (Shestakov et al., 2019; Jiang et al., 2014; Ran et al., 2017). The membranes for use in EED can either be 1) cationic membranes that have negative fixed groups with positive mobile ions that allow movement of cations or 2) anionic membranes that have positive fixed groups and against negative mobile ions that allow the passage of anions through it.

Widespread research interests regarding applications of EED are being directed toward applications such as water purification (Wu et al., 2019; Mejía et al., 2020), organic and inorganic acid and base recovery (Chapotot et al., 1995; Yi et al., 2008; Wei et al., 2013; Tanaka et al., 2014; Miao et al., 2016; Pisarska et al., 2017), as well as metal concentration from liquid sources (Jin et al., 2016; Liu et al., 2020). Par excellence, electrochemical processes have been chosen as efficient, effective, and reliable methodologies for processes involving the selective recovery of metallic species (Jin and Zhang, 2020). Among techniques such as electrosorption, electrodeionization, and electrocoagulation, the seamless combination of electrodialysis and electrodeposition in EED provides good performance as well as is being cost efficient. Interesting discussions can be found in the literature regarding the economic aspects of the scaling of the EED techniques, bases on energy costs, process capacity, peripheral equipment, membrane durability, maintenance, *etc*. (Wei et al., 2013).

From the aforementioned technical and productive perspective, it is interesting to explore the behavior of the copper/ammonia system proposed, which can be approached from two main viewpoints, namely, the efficiency of copper recovery and the quality of the deposits obtained, and second, to determine the extent of the ammonia required for the process to run properly, as well as its reusability. From this, the principal objective of this scientific work is to study the quality of the resulting copper cathode when the system is working with different copper concentrations in solutions with excess ammonia, under different current densities tested in an EED cell.

## Experimental

**FIGURE 1:**
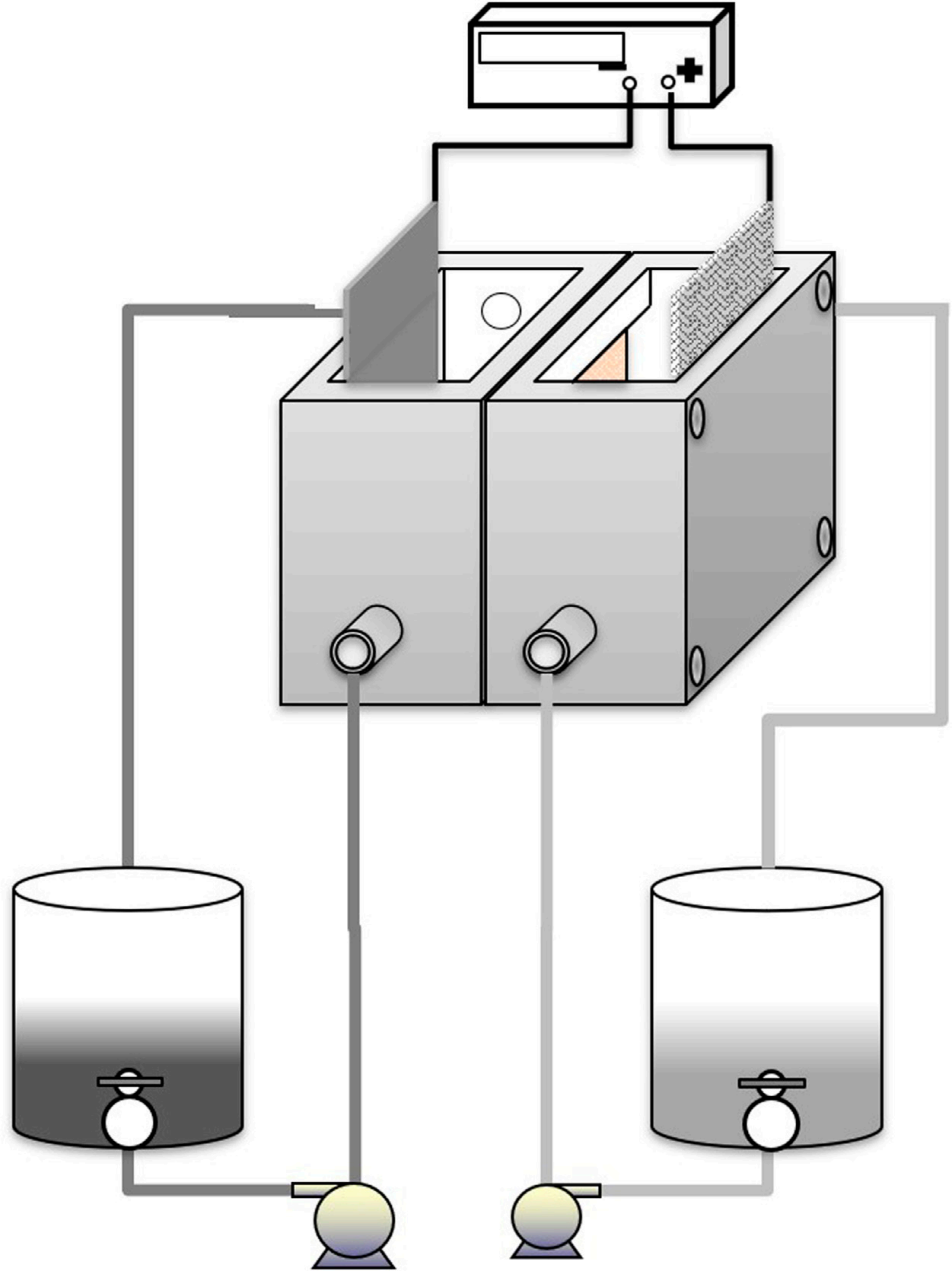
Scheme of the systems used in the EED experiments.

Figure 1 shows a general schematic representation of the electrolytic circuit at the laboratory level where the process of electro obtaining copper is carried out by electro-electrodialysis. The acrylic cell has two compartments of 125 cm^3^ (0.125 dm^3^), and a cation exchange type membrane was used (Ionsep HC-C, area 0.25 dm^2^) between the electrodes, at a distance of 10 cm, separating anolyte and catholyte.

The catholyte (1 dm^-3^ volume) contains a solution of cupric tetramine at pH = 9.0, and the anolyte (1 dm^−3^ volume) contains a solution of NaOH at pH = 12.5; catholyte and anolyte were distributed in each compartment using pumps. Electrolytes were fed from the bottom, and discharge was carried out from above by gravity, while the recirculation flow of both electrolytes remained constant at 2 dm^−3^ min^−1^. The cell operated at normal pressure and temperature conditions. A stainless steel cathode was used with an effective area of 0.04 dm^2^ and an anode with titanium mesh coated with ruthenium oxide was used. The oxidation reaction of hydroxyl ions occurs in the anode compartment. Finally, the EED test was carried out for 3 h, and cupric ion (Cu^+2^) concentrations ranging from 0.01 to 0.1 mol dm^−3^ were studied, at different current density values, from 200 to 500 A m^−2^.

## Results and Discussion

The copper recovery process as a cathodic deposit by EED is dependent on several factors that theoretically determine its electrodeposition at the cathode: among them are electrode nature, ion exchange membrane, current density, temperature, volumetric flow, working time, and pH of the electrolyte (Audinos, 1986; Shestakov et al., 2019).

To study the effect of the electro-active species concentration on deposit morphology, a series of experiments were carried out. The cupric ion concentration was varied from 0.01 to 0.10 mol dm^−3^, with a constant current density of 200 A m^−2^. There is a relationship between the current density 1) and the cathodic overpotential (η) in the mixed control range (charge transfer and diffusion) represented by Eq. 1.

$$i_{c}=i_{o,c}\left[\frac{C_{o}^{s}}{C_{o}}\exp\left(\frac{\alpha_{c}n\;F}{RT}\left(-\eta \right)\right)\right]$$ (1)

where i_c_ is the cathodic current density (A·m^−2^), i_o,c_ is the exchange current density (A·m^−2^), C_0_ is the concentration of the electro-active species in solution bulk (mol·dm^−3^), C_0_^S^ is the concentration of the electro-active species on the electrode surface (mol·dm^−3^), *η* is the cathodic overpotential (*V*), *α*_c_ is the cathodic transfer coefficient, Faraday constant (F; 96,500 C·eq^−1^), n is the number of electrons transferred in the reaction (eq·mol^−1^), R is the universal gas constant (8.314 J mol^−1^ K^−1^), and *T* is the operating temperature (*K*).

**FIGURE 2:**
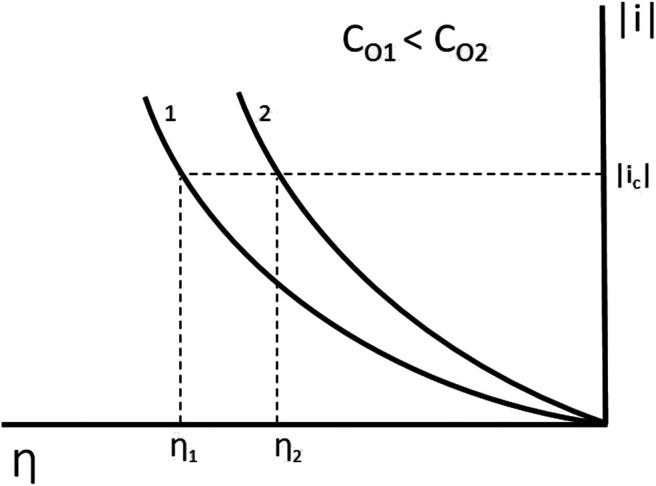
Graphical representation of potential variation on different characteristics of the electro-active species.

Taking into consideration from Eq. 1 that exchange current density and charge transfer coefficient are inherent parameters of a system, it is observed that for a constant current density, as the concentration of electro-active species increases on the bulk solution, the cathodic overpotential decreases, as seen in Figure 2.

**FIGURE 3:**
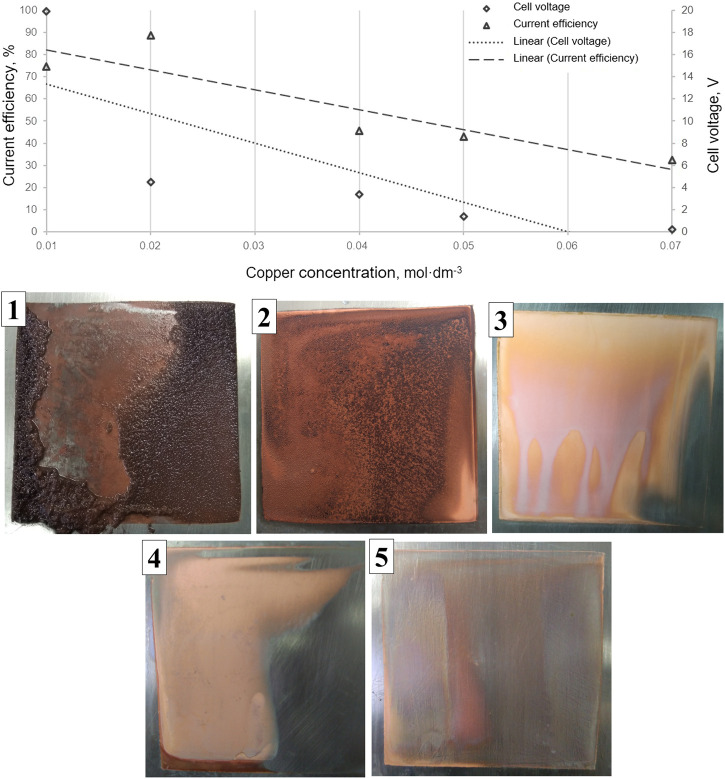
**(Above)** Current efficiency and cell voltage in copper EED depending on the concentration of the electro-active specie at 200 A m^−2^. Data: (◊) cell voltage; (Δ) current efficiency. Dotted and segmented lines represent the fit of the data corresponding to the cell voltage and current efficiency, respectively. **(Below)** Photographs (1–5) of the respective cathode deposits, at the different Cu^+2^ concentrations (from lower to higher) specified for tests EED1–EED5 in Table 1.

The morphology of the metallic deposits depends mainly on the kinetic parameters and the cathodic overpotential or current density (Guo and Searson, 2010; Nagar et al., 2013; González Mercado et al., 2015). Similarly, the nucleation overpotential ($\eta_{n}$), defined as the potential corresponding to the onset of deposition for the reaction $M^{+}+e^{-}\to M^{0}$, has important implications for island growth and the evolution of surface morphology (Guo and Searson, 2010). On the other hand, an increase in Cu^+2^ concentration results in lower nucleation overpotential. Such as the deposit five in Figure 3, the deposition overpotential can be controlled not only by the applied current density but also by changes in Cu^+2^ concentration, so at a constant current intensity, copper deposition overpotential decreases. This can be observed in picture five insert on Figure 3, evidencing the slight growth produced despite working at low deposition overpotential.

**TABLE 1:** Specific operational parameters used for the EED tests.

Test	[Cu^+2^] (mg dm^−3^)	Current density (A·m^−2^)	Test	[Cu^+2^] (mg dm^−3^)	Current density (A·m^−2^)
EED1	0.01	200	EED6	0.10	200
EED2	0.02	200	EED7	0.05	100
EED3	0.04	200	EED8	0.05	300
EED4	0.05	200	EED9	0.05	400
EED5	0.07	200	EED10	0.05	500

On the contrary, for a low Cu^+2^ concentration, the nucleation overpotential increases along with the deposition overpotential. This causes a considerable increase of an irregular surface and the subsequent growth of these irregularities. Consequently, it is possible to obtain dendrites and dispersed deposit morphology without privileged orientation when working at lower copper concentrations (see metal deposit one in Figure 3).

Figure 3 shows the variation in current efficiency and cell voltage as a function of the electro-active specie concentration on the bulk solution (tests EED1-EED5, in EED6, the deposit is not evidenced) where a decrease is observed for both current efficiency and voltage with the increase in Cu^+2^ concentration (keeping current density constant at 200 A m^−2^) associated by a decrease in both the nucleation overpotential and the deposition overpotential, respectively. As a comparison, for the cell voltage, a significative decrease is observed at low concentrations of Cu^+2^, ranging from 20 to 4 V at 0.02 mol dm^−3^, corresponding to a five-fold change in the applied potential to the operating cell, followed by a relatively stable potential observed in the Cu^+2^ concentration interval between 0.02 and 0.05 mol dm^−3^. Photographs one through five in Figure 3 show the progression of the morphology of the copper deposit with the increase in Cu^+2^ concentration in the EED cell, where the change from irregular granular deposits at low Cu^+2^ concentrations to a smooth-surfaced, ordered deposits at higher Cu^+2^ concentrations can be observed.

**FIGURE 4:**
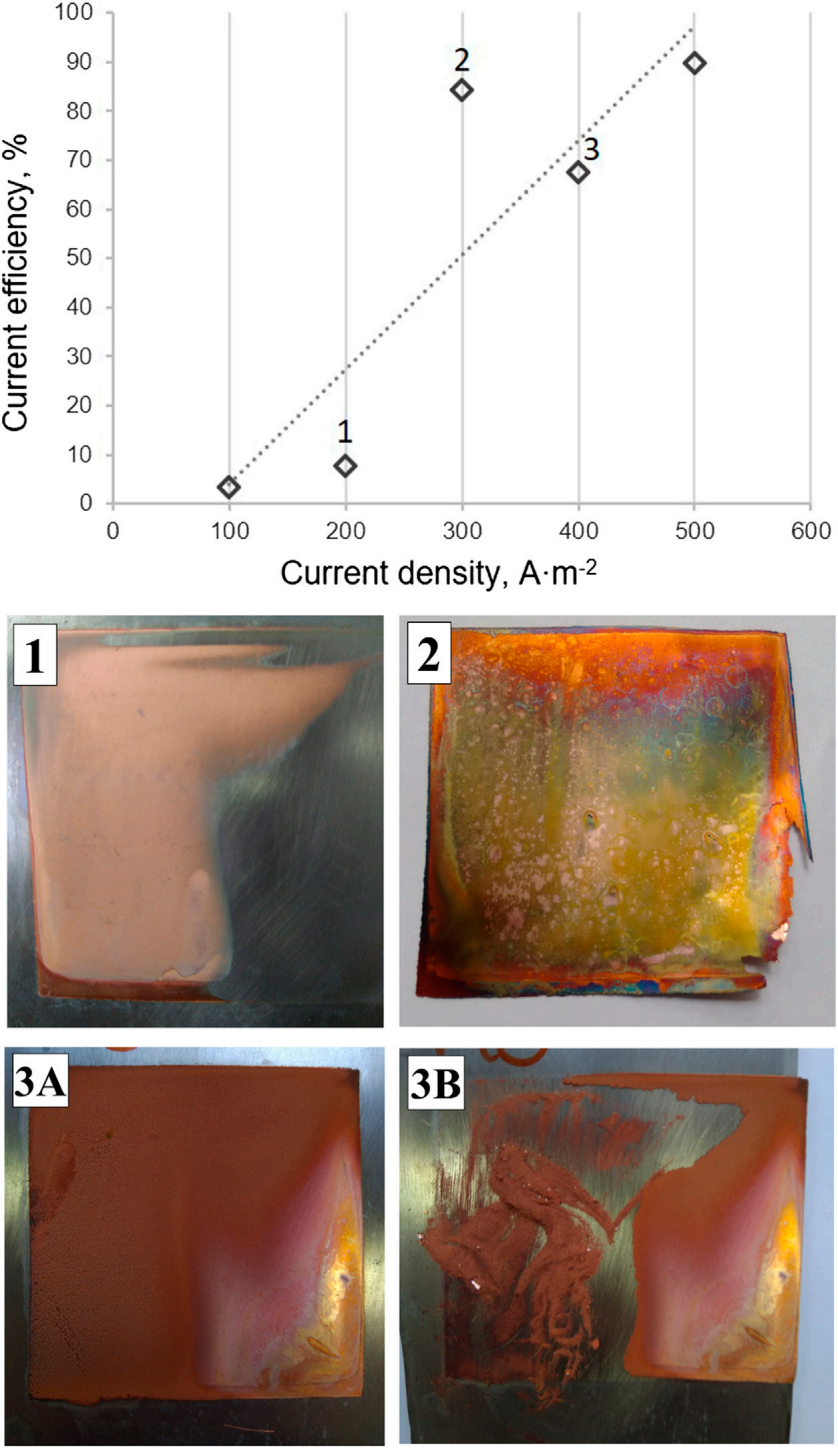
**(Above)** Current efficiency as function of the current density applied for solution of 0.05 mol dm^−3^ copper and stoichiometric ammonia. **(Below)** Photographs of the respective cathode deposits, for tests EED4 and EED7–EED10 in Table 1. Pictures 3A and 3B correspond to the same cathode deposit before and after the scraping of the deposited copper.

To study the effect of current density in the process, tests were carried out at 100, 200, 300, 400, and 500 A m^−2^ with a copper solution at a constant concentration equal to 0.05 mol dm^−3^. The theory proposes that as an increase in current density leads to an increase in overpotential, more nucleation sites are activated at higher current densities (Guo and Searson, 2010). The behavior proposed by this theory is observed in Figure 4 (test EED 4, 7, 8, 9, and 10) where a linear increase in current efficiency of the system is observed with current density at the experimental condictions tested for the copper deposition experiments, caused by the increased metal deposit mass but with a poor substrate quality (see pictures 1, 2, 3A, and 3B in Figure 4). This is due to high deposit overpotential achieved with a high frequency of nucleation over grain growth, causing a deposit of dispersed crystals without privileged orientation or UD in the Fisher classification (Nagar et al., 2013; Markov, 2016).

On the other hand, ammonia plays an important role in the process since it is involved in the formation of cupric ion complexes in the copper leaching such as [Cu (NH_3_)_4_]^2+^; in addition, its concentration influences the presence of other unwanted substances, such as copper oxide. Likewise, there is a constant competition between ammonia and the overpotential of deposition for keeping copper in solution and reducing it in the cathode, respectively.

**FIGURE 5:**
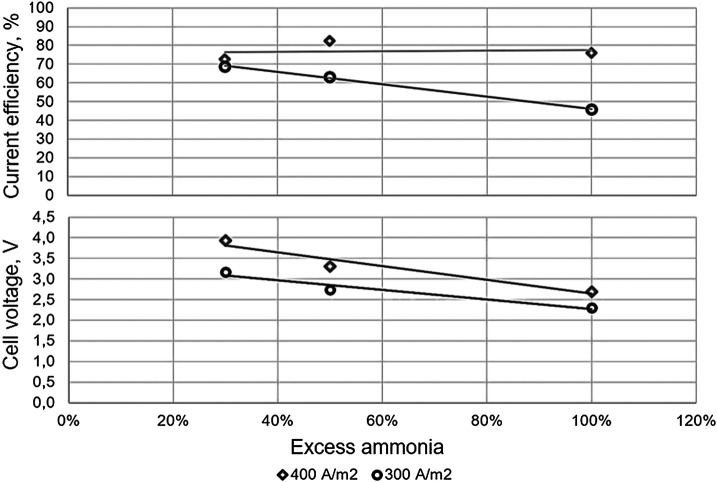
Current efficiency **(above)** and cell voltage **(below)** as function of excess ammonia for solution 0.05 mol dm^−3^ copper to 300 (◯) and 400 (◇) A m^−2^.

To study the effect of ammonia on the system, excess ammonia (30, 50, and 100% excess, in reference to the stoichiometric ratio with copper) were added to the solutions, and two different current densities 300 and 400 A m^−2^ were operated. Consequently, a low current efficiency is obtained when operating with higher concentrations of NH_3_ (ammonia) as an excess, at the highest current density considered, as seen in Figure 5, where it can be seen that, at the highest current density (400 A m^−2^), no variation is observed from the current efficiency perspective with the increase in excess ammonia concentration, contrary to what is observed at the lowest current density considered (300 A m^−2^), where almost a two-fold decrease is observed in the current efficiency with a three-fold increase in excess ammonia.

In addition, a proportional decrease in cell voltage with the increase of excess ammonia is observed at both of the current density values considered, given that, in both conditions, almost a 30% decrease is observed in the cell voltage with the increase of ammonia concentration. The observed behavior is mainly due to the increase of ions in solution, as hydroxyl and ammonium ions that, in turn according to conditions, would form ammonia and protons according to reaction in Eq. 2,

$${\text{NH}}_{3}{+\text{H}}^{+}⇆\;{\text{NH}}_{4}^{+}$$ (2)

causing an increase in conductivity, due to a decrease in resistance to the passage of current; therefore, a voltage drop is observed.

In relation to variation in current density, results were consistent in relationship to the theory discussed above, where concomitant to a higher current density, a higher cell voltage is observed due to the increase in overpotential; and a higher current efficiency is caused by an increase in copper deposited mass, which is not observed in current copper industry in sulfuric medium.

## Conclusion

It is feasible to carry out copper electrowinning in a cell based on EED applying high current density at low catholyte copper concentration, 3 g L^−1^ and at pH = 9. The oxidation reaction at the anode with an aqueous alkaline medium as anolyte at pH = 12.7 was the water oxidation.

In all cases, current efficiency increases as current density increases; however, at a current density above 400 A⋅m^−2^, a deposit of low adhesion and fine size was obtained, conditions that occur when the system was operating near to the limit current density. For concentrations of 0.05 mol dm^−3^ of Cu^+ 2^, the current efficiency increases by 130%, from 200 to 500 A⋅m^−2^. The cell voltages obtained were in the range of 2–4 V, not far above conventional processes (2.2 V), and power consumption of 0.1–0.7 W.

In summary, it was possible to develop a novel process for the electrochemical deposition of copper from an ammoniacal copper catholyte using cationic ion exchange membranes to prevent the destruction of complexing agent NH_3_/NH^4+^, which can be reused in ammoniacal leaching.

## Data Availability

The original contributions presented in the study are included in the article/Supplementary Material, and further inquiries can be directed to the corresponding author.
